# The relationship between chronic expansion of self-expandable valves and paravalvular leakage in transcatheter aortic valve implantation

**DOI:** 10.1007/s12928-025-01140-7

**Published:** 2025-05-20

**Authors:** Yuuki Muto, Daisuke Isomatsu, Yu Sato, Akihiko Sato, Takeshi Shimizu, Tomofumi Misaka, Masayoshi Oikawa, Atsushi Kobayashi, Akiomi Yoshihisa, Kazuhiko Nakazato, Takafumi Ishida, Hirofumi Sekino, Kenji Fukushima, Hiroshi Ito, Yasuchika Takeishi

**Affiliations:** 1 Department of Cardiovascular Medicine, Fukushima Medical University, 1 Hikarigaoka, Fukushima, 960-1295 Japan; 2 Department of Radiology and Nuclear Medicine, Fukushima Medical University, Fukushima, Japan

**Keywords:** Aortic stenosis, Transcatheter aortic valve implantation (TAVI), Paravalvular leakage (PVL), Valve expansion, Computed tomography (CT)

## Abstract

Paravalvular leakage (PVL) is a clinical concern in transcatheter aortic valve implantation (TAVI). Although PVL has been reported to gradually decrease after self-expandable valves (SEVs) implantation, the mechanism remains unclear. Furthermore, while gradual expansion of SEVs has been documented, studies assessing their morphological changes during the chronic phase are lacking. This study aims to evaluate whether SEVs expansion occurs during follow-up and if this expansion correlates with a reduction in PVL. We included 60 patients who underwent TAVI using SEVs (Evolut PRO/PRO+/FX) from October 2020 to August 2024. Morphological findings of SEVs were assessed using computed tomography (CT), and PVL was evaluated at discharge and chronic phase post-TAVI. PVL was graded on a five-point scale via transthoracic echocardiography, and the area ratio, defined as the ratio of the basal area of the implanted SEVs to that of phantom valves measured by CT, served as the index of SEVs expansion. Chronic expansion of SEVs was observed in all cases, with the area ratio increasing from 0.59 at discharge to 0.64 at the follow-up (*P* < 0.05). Among the 60 patients, 44 exhibited mild or greater PVL, with 17 showing improvement at the chronic phase. The degree of valve expansion (area ratio at chronic phase/area ratio at discharge) was significantly greater in the PVL improvement group compared to the non-improvement group (1.11 vs. 1.06, *P* < 0.05). This study demonstrates that SEVs expand during the chronic phase after TAVI, which could contribute to the reduction of PVL.

**Graphical abstract:**

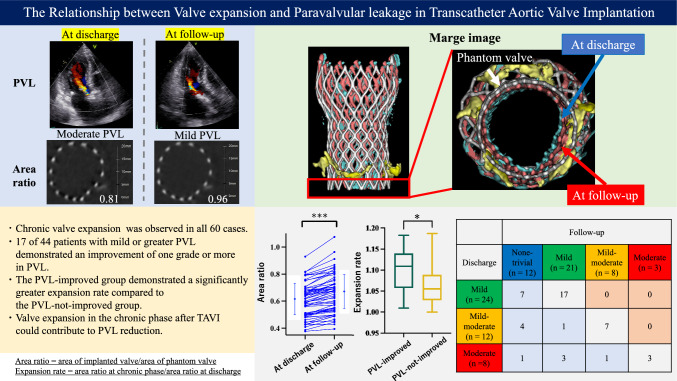

## Introduction

Transcatheter aortic valve implantation (TAVI) has increasingly expanded its indications as a standard treatment for aortic valve stenosis [1, 2]. As the indications for TAVI have expanded to include younger and lower surgical risk patients [1, 2] lifetime management focusing on the long-term outcome following TAVI has become increasingly important. Paravalvular leakage (PVL) is one of the complications associated with TAVI that can impact long-term prognosis [3]. Moderate or greater PVL has been reported to affect prognosis [3], and even mild PVL can also influence outcomes [4], making it a crucial complication to consider when evaluating long-term results. Self-expandable valves (SEVs) are noted for their minimal risk of annular rupture, a fatal complication in TAVI [5]. However, they are known to be associated with a higher incidence of PVL compared to balloon-expandable valves (BEVs) [5]. While PVL remains a significant challenge in TAVI procedures utilizing SEVs despite advancements in device technology [6, 7], SEVs are reported to expand immediately post-TAVI [8], and some studies have suggested a potential decrease in PVL during the chronic phase [9–11]. However, the extent to which SEVs expand over time and how this expansion contributes to the reduction in chronic-phase PVL are not yet fully understood. Therefore, the aim of this study was to evaluate whether expansions of SEVs were observed during follow-up and to investigate if its expansion was associated with a reduction in PVL.

## Methods

### Study protocol and patients’ information

This single-center prospective observational study included consecutive 60 patients who underwent TAVI with SEVs [Evolut PRO/PRO+/FX (Medtronic Inc., Minneapolis, MN, USA)] at our institution from October 2020 to August 2024. Cases involving bicuspid valves and TAVI in previously surgically replaced valves were excluded. All 60 patients were assessed for PVL using transthoracic echocardiography and for calcification of the aortic valve complex and morphology of the SEVs using non-contrast computed tomography (CT) at discharge and at chronic phase ranging 3 months to 1 year after TAVI. Since the follow-up was conducted based on the attending physician's judgment, there were actually some variations in the follow-up period (mean follow-up 196.6 ± 107.8 days).”

**Fig. 1:**
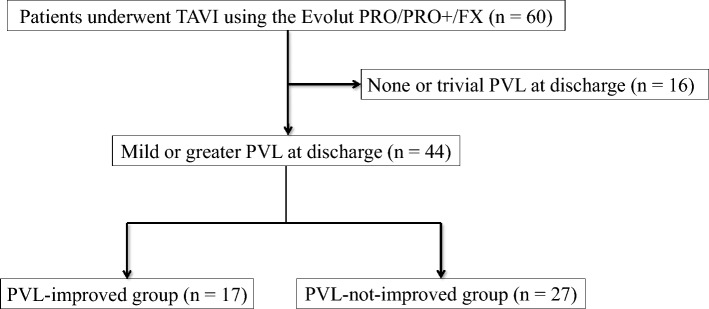
Flowchart of patient selection

Among the cases with mild or greater PVL identified at discharge, patients were divided into two groups based on whether they showed improvement in PVL at the chronic phase follow-up: the PVL-improved group and the PVL-not-improved group (Fig. 1). Analyses were then conducted to evaluate changes in valve morphology during the chronic phase between the two groups. Comorbidities were evaluated by several attending physicians using medical records [12].

The study protocol was approved by the Ethics Committee of Fukushima Medical University (approval number, C-T2022-0348), and was carried out in accordance with the principles outlined in the Declaration of Helsinki [13]. Reporting of this study conforms to the Strengthening the Reporting of Observational Studies in Epidemiology guidelines [14].

### CT image analysis

CT scans were conducted with a 320-detector-row CT scanner (Aquilion Prime SP, Canon Medical Systems Corporation, Otawara, Japan). Aortic root assessments were performed by analyzing pre-TAVI CT images using Ziostation2 (Ziosoft Inc., Tokyo, Japan).

After identification of the basal plane, the aortic root was divided into the following regions in a multiplane image: the non-coronary cusp (NCC), the right-coronary cusp (RCC), and the left-coronary cusp (LCC). Each cusp was manually delineated to obtain the correct boundary, and the calcification below the basal plane to 5 mm into the left ventricle was defined as calcification of the left ventricular outflow tract (LVOT). Other calcified sites such as the proximal coronary artery were manually excluded. As previously reported, the automated volume measurement tool (Ziostation2) was used to quantify the volume with an empiric threshold cut-off value of 550 Hounsfield units [15]. The oversizing rate was calculated as: (transcatheter heart valve [THV] nominal perimeter-annulus perimeter/annulus perimeter) × 100.

**Fig. 2:**
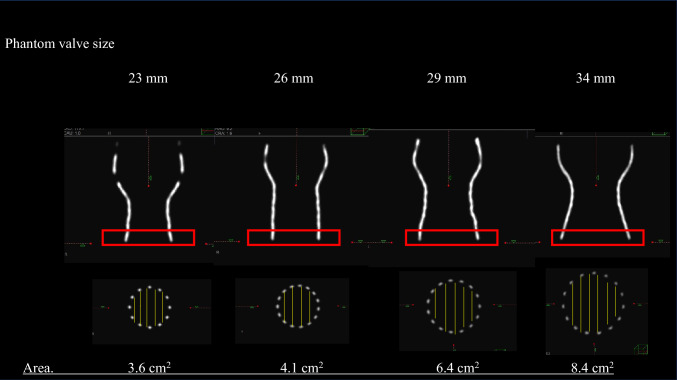
Methods of measuring basal area of the phantom valves

The evaluation of valve morphology was conducted by comparing and analyzing the phantom valves of various sizes with the implanted valves, using CT imaging. Medtronic Inc. provided Evolut PRO+ phantom valves in sizes 23 mm, 26 mm, 29 mm, and 34 mm and they ware imaged using CT to obtain 3D reconstructed images. The basal area of valves was defined as an index of valve expansion, and the basal area of the phantom valves was measured to establish control values (23 mm, 3.6 cm^2^; 26 mm, 4.1 cm^2^; 29 mm, 6.4 cm^2^; 34 mm, 8.4 cm^2^) as shown in Fig. 2. After TAVI procedure, for each case, electrocardiogram-gated plane CT was performed at discharge and chronic phase to construct 3D images of the implanted valves and measure their basal area. The ratio of the basal area of the phantom valves to that of the implanted valves was calculated as the area ratio. Additionally, the ratio of the basal area of the implanted valves at discharge to that at chronic phase after TAVI was defined as the expansion rate, serving as an index of chronic valve expansion.

### PVL assessment

PVL was evaluated according to the VARC-3 criteria using transthoracic echocardiogram (TTE) on a five-point scale (none or trivial, mild, mild-moderate, moderate, severe), with mild or greater defined as significant PVL and two-dimensional color Doppler images from the three-chamber view of TTE were used for PVL assessment [16, 17]. TTE was performed at discharge and chronic phase post-procedure to evaluate PVL, with cases demonstrating an improvement of one grade or more defined as having PVL improvement. Echocardiograms were acquired using three ultrasound machines (Vivid E95, General Electric Healthcare, Milwaukee, WI, USA; EPIQ7, Philips Healthcare, Amsterdam, the Netherlands; and Aplio700, Canon Medical Systems, Tokyo, Japan).

### TAVI procedure and post-TAVI management

Severe AS was defined as an aortic valve area of < 1.0 cm^2^ and a mean pressure gradient of > 40 mmHg or peak velocity of > 4.0 m/s on transthoracic echocardiography [18]. Decisions regarding TAVI, including the choice of access route (transfemoral approach as the first choice) as well as the type and size of prosthetic valves, were made based on the results of CT analysis by consensus of the multidisciplinary heart team at Fukushima Medical University Hospital, comprising senior interventional cardiologists, cardiovascular surgeons, and imaging specialists. TAVI was performed under general anesthesia in a hybrid catheterization laboratory. Decisions regarding whether to perform pre-dilation and post-dilation were left to the discretion of the heart team, generally based on the findings of significant PVL on post-deployment echocardiographic imaging. Using a cusp-overlap view, a SEV (Evolut PRO/PRO+ and FX, Medtronic Inc.) was implanted 3–5 mm below the annulus [19]. Antithrombotic therapy after TAVI consisted of a single antiplatelet, double antiplatelet, or anticoagulant agent. The patients were monitored in the cardiac care unit for several hours after the procedure. After being transferred to the general ward, the patients received comprehensive cardiac rehabilitation and guideline-based medical therapy [18].

### Statistical analysis

Data were analyzed using SPSS version 28 (SPSS, Armonk, NY, USA). *P* values of < 0.05 were considered statistically significant for all analyses. The normality of each group was verified by a Shapiro–Wilk test. Parametric variables were presented as mean ± standard deviation, and non-parametric variables were presented as a median with interquartile range. Parametric and non-parametric variables were compared using Student's *t* test, and Mann–Whitney *U* test, respectively. Categorical variables were expressed as numbers and percentages, and the Chi-square test was used for comparison. To assess the effectiveness of the intervention, a paired *t* test was conducted between the pre- and post-TAVI data.

## Results

The baseline data for the 60 analyzed patients are presented in Table 1. Among the treated cases, 53.3% were male, and the mean BMI was 24.5 ± 4.1 kg/m^2^. The parameters for aortic stenosis included a peak aortic valve velocity of 4.9 ± 0.7 m/s, a mean aortic valve pressure gradient of 57.5 ± 16.0 mmHg, and an aortic valve area of 0.65 ± 0.20 cm^2^. The preoperative aortic regurgitation was 6.7%, 70.0%, and 23.3% at none/trivial, mild, and moderate, respectively. The left ventricular ejection fraction was 63.0% [interquartile (IQR) 57.8–68.0], and the B-type natriuretic peptide (BNP) level was 282.6 pg/ml [IQR 79.1–474.6]. All TAVI procedures were performed via a transfemoral approach. Pre-dilation was conducted in 57 cases, and the cusp-overlap technique was used for valve deployment, achieving a procedural success rate of 100%. The distribution of implanted valves included 5 cases of 23 mm, 27 cases of 26 mm, 27 cases of 29 mm, and 1 case of 34 mm.

**Table 1:** Baseline characteristics (*n* = 60)

	Total (*n* = 60)
Age (years)	84.0 ± 4.9
Male gender (*n*, %)	32 (53.3)
Smoker (*n*, %)	20 (33.3)
BMI (kg/m^2^)	24.5 ± 4.1
Comorbidity	
Hypertension (*n*, %)	47 (78.3)
Diabetes mellitus (*n*, %)	14 (23.3)
Dyslipidemia (*n*, %)	34 (56.7)
CKD (*n*, %)	35 (58.3)
Atrial fibrillation (*n*, %)	11 (18.3)
LEAD (*n*, %)	3 (5.0)
Stroke (*n*, %)	6 (10.0)
Laboratory data before TAVI	
eGFR (ml/min/1.73 m^2^)^a^	54.9 ± 14.6
Hemoglobin (g/dl)^a^	12.1 ± 1.5
BNP (pg/ml)^a^	282.6 (79.1, 474.6)
Echocardiographic variables before TAVI	
LVEF (%)^a^	63.0 (57.8, 68.0)
Aortic valve area (cm^2^)	0.65 ± 0.20
Mean aortic valve pressure gradient (mmHg)	57.5 ± 16.0
Max aortic valve pressure gradient (mmHg)	96.1 ± 26.4
Peak aortic jet velocity (m/s)	4.9 ± 0.7
Stroke volume (ml)	73.9 ± 18.2
Aortic regurgitation	
None/trivial, (*n*, %)	4 (6.7)
Mild, (*n*, %)	42 (70.0)
Moderate, (*n*, %)	14 (23.3)
Severe, (*n*, %)	0 (0.0)
CT variables before TAVI	
Annulus area (mm^2^)	417.6 ± 76.9
Circumference of valves (mm)	74.0 ± 6.8
Ca-Vol (cm^3^)	2.4 ± 1.0
LVOT calcification (*n*, %)	15 (25.0)
Procedural variables	
Valve size: 23 mm, (*n*, %)	5 (8.3)
Valve size: 26 mm, (*n*, %)	27 (45.0)
Valve size: 29 mm, (*n*, %)	27 (45.0)
Valve size: 34 mm, (*n*, %)	1 (1.7)
Implantation depth (mm)	4.0 ± 1.6
Pre-dilation (*n*, %)	57 (95.0)
Post-dilation (*n*, %)	5 (8.3)
Pre-dilation size (mm)	21.6 ± 2.6
Medications at discharge	
Beta-blocker	8 (13.3)
Diuretics	18 (30.0)
ACEi/ARB	26 (43.3)

BMI, body mass index; CKD, chronic kidney disease; LEAD, lower extremity artery disease; eGFR, estimated glomerular filtration rate; BNP, B-type natriuretic peptide; LVEF, left ventricular ejection fraction; LVOT, left ventricular outflow tract; Ca-Vol, calcium volume; ACEi, angiotensin-converting enzyme inhibitor; ARB, angiotensin II receptor blocker

^a^Median (interquartile range)

**Fig. 3:**
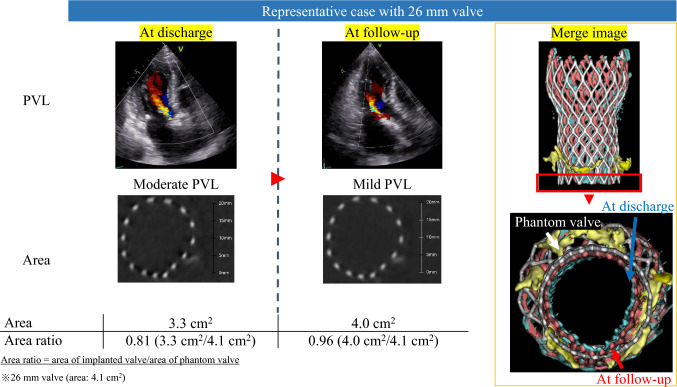
Representative case. This case presented an area of an implanted 26 mm valve and PVL. Valve expansion and PVL decrease were observed at follow-up. The merged images of the phantom valve (white), the valve at discharge (blue), and the valve at follow-up (red) are shown. Valve expansion was noted from discharge to follow-up

We present a case of PVL that improved during the chronic phase (Fig. 3). This patient underwent TAVI with a 26 mm Evolut PRO+ valve and was found to have moderate PVL at discharge. Follow-up echocardiography revealed an improvement to mild PVL. CT analysis indicated that the area ratio at discharge was 0.81 (with the basal area of the implanted valve measuring 3.3 cm^2^ and the basal area of the phantom valve measuring 4.1 cm^2^). At the follow-up, the area ratio improved to 0.96 (with the basal area of the implanted valve measuring 4.0 cm^2^), resulting in an expansion rate of 1.18, demonstrating chronic expansion of the implanted valve.

**Fig. 4:**
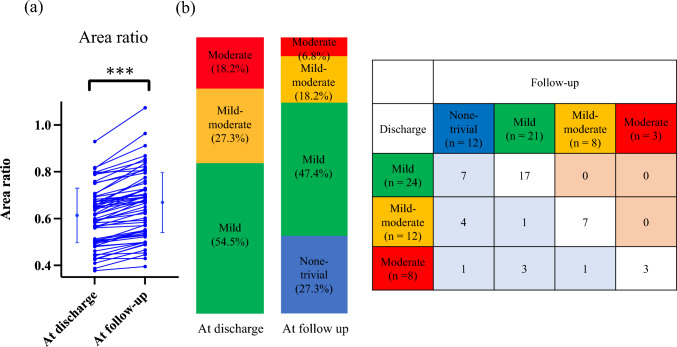
Changes in area ratio (**a**) and degree of PVL from discharge to follow-up (**b**). The area ratio increased and PVL improved at follow-up. ***P < 0.001

The results of the valve morphology assessment via CT indicated that the implanted valves expanded in all 60 cases at the chronic phase compared to discharge (Fig. 4a). At discharge, echocardiography revealed that 44 patients had mild or greater PVL, including 24 cases (54.5%) classified as mild, 12 cases (27.3%) as mild-moderate, and 8 cases (18.2%) as moderate (Fig. 4b). During the follow-up, 17 of these 44 cases demonstrated an improvement of one grade or more in PVL.

**Fig. 5:**
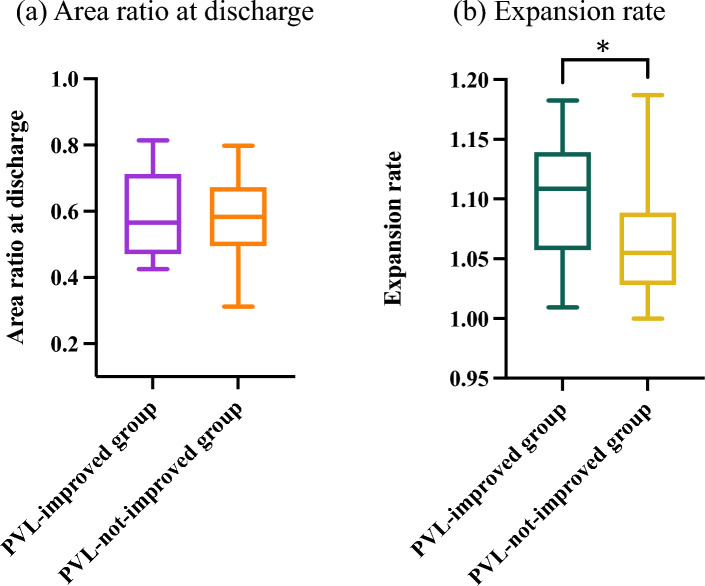
Comparisons of area ratio at discharge (**a**) and expansion rate between the PVL-improved group and the PVL-not-improved group (**b**). Expansion rate was larger in PVL-improved group than in PVL-not-improved group. *P < 0.05

The baseline data for the PVL-improved group (*n* = 17) and the PVL-not-improvement group (*n* = 27) are presented in Table 2. There were no differences between the two groups in terms of sex, age, and BMI. Additionally, no significant differences were observed in the severity of aortic stenosis and aortic regurgitation or the amount of calcification in the aortic valve complex. In comorbidity, hypertension was more prevalent in the PVL-not-improvement group. Regarding procedural factors, duration of the procedure, contrast volume, proportions of pre-dilation and post-dilation, valve deployment depth, distribution of implanted valve sizes, LVOT calcification, and oversizing rate were similar between the two groups. Medications, such as beta-blockers, diuretics, angiotensin-converting enzyme inhibitor (ACEi), and angiotensin II receptor blocker (ARB) at discharge, did not differ between the two groups. There was no significant difference in the area ratio at discharge between the two groups (PVL-improved group: 0.59 ± 0.13, PVL-not-improved group: 0.58 ± 0.13, *P* = 0.715) as shown in Fig. 5a. However, the PVL-improved group demonstrated a significantly greater expansion rate (1.11 [IQR 1.06–1.14] vs. 1.06 [IQR 1.03–1.09], *P* = 0.023) as demonstrated in Fig. 5b.

**Table 2:** Baseline characteristics (*n* = 44)

	Total (*n* = 44)	PVL-improved group (*n* = 17)	PVL-not-improved group (*n* = 27)	*P* value
Age (years)	84.3 ± 5.3	84.2 ± 4.3	84.4 ± 5.8	0.886
Male gender (*n*, %)	21 (47.7)	9 (56.2)	12 (42.9)	0.392
Smoker (*n*, %)	17 (38.6)	5 (31.2)	12 (42.9)	0.447
BMI (kg/m^2^)	24.5 ± 3.8	23.8 ± 3.0	24.9 ± 4.2	0.369
Comorbidity				
Hypertension (*n*, %)	31 (70.5)	8 (47.1)	22 (81.5)	0.043
Diabetes mellitus (*n*, %)	7 (15.9)	1 (6.2)	6 (21.4)	0.188
Dyslipidemia (*n*, %)	24 (54.5)	6 (37.5)	18 (64.3)	0.086
CKD (*n*, %)	27 (61.4)	8 (47.0)	19 (67.9)	0.242
Atrial fibrillation (*n*, %)	7 (15.9)	4 (23.5)	3 (10.7)	0.205
LEAD (*n*, %)	3 (6.8)	0 (0.0)	3 (10.7)	0.247
Stroke (*n*, %)	3 (6.8)	0 (0.0)	3 (10.7)	0.247
Laboratory data before TAVI				
eGFR (ml/min/1.73 m^2^)^a^	53.7 ± 13.8	55.7 ± 14.1	52.5 ± 13.8	0.476
Hemoglobin (g/dl)^a^	11.9 ± 1.5	11.8 ± 1.5	12.0 ± 1.5	0.683
BNP (pg/ml)^a^	310.9 (117.8, 500.6)	373.9 (152.9, 541.1)	290.0 (117.8, 495.9)	0.617
Echocardiographic variables before TAVI				
LVEF (%)^a^	62.5 (56.8, 67.0)	60.5 (56.0, 66.5)	63.0 (57.8, 67.0)	0.874
Aortic valve area (cm^2^)	0.63 ± 0.21	0.61 ± 0.21	0.64 ± 0.21	0.700
Mean aortic valve pressure gradient (mmHg)	59.0 ± 15.2	62.9 ± 16.7	56.7 ± 14.2	0.193
Max aortic valve pressure gradient (mmHg)	97.8 ± 24.3	101.8 ± 28.0	95.6 ± 22.2	0.427
Peak aortic jet velocity (m/s)	4.9 ± 0.6	5.0 ± 0.7	4.9 ± 0.6	0.422
Stroke volume (ml)	72.7 ± 19.4	72.1 ± 17.3	73.1 ± 20.8	0.878
Aortic regurgitation				0.139
None/trivial, (*n*, %)	3 (6.8)	0 (0.0)	3 (11.1)	
Mild, (*n*, %)	32 (72.7)	12 (70.6)	20 (74.1)	
Moderate, (*n*, %)	9 (20.5)	5 (29.4)	4 (14.8)	
Severe, (*n*, %)	0 (0.0)	0 (0.0)	0 (0.0)	
CT variables before TAVI				
Annulus area (mm^2^)	434.1 ± 77.1	442.7 ± 93.1	429.3 ± 67.8	0.585
Circumference of valves (mm)	75.5 ± 6.6	76.2 ± 7.6	75.1 ± 6.0	0.597
Ca-Vol (cm^3^)	2.7 ± 0.9	2.6 ± 0.8	2.7 ± 0.9	0.474
LVOT calcification				0.880
Mild, (*n*, %)	4 (9.1)	2 (11.8)	2 (7.4)	
Moderate, (*n*, %)	5 (11.4)	2 (11.8)	3 (11.1)	
Severe, (*n*, %)	0 (0.0)	0 (0.0)	0 (0.0)	
Oversizing rate (%)	17.9 ± 5.3	17.0 ± 4.6	18.6 ± 5.7	0.350
Procedural variables				
Valve size				0.397
23 mm, (*n*, %)	2 (4.5)	1 (5.9)	1 (3.7)	
26 mm, (*n*, %)	19 (43.2)	8 (47.1)	11 (40.7)	
29 mm, (*n*, %)	22 (50.0)	7 (41.2)	15 (55.6)	
34 mm, (*n*, %)	1 (2.3)	1 (5.9)	0 (0.0)	
Implantation depth (mm)	4.2 ± 1.6	4.3 ± 1.2	4.1 ± 1.8	0.695
Pre-dilation (*n*, %)	43 (97.7)	16 (94.1)	27 (100.0)	0.636
Post-dilation (*n*, %)	5 (11.4)	2 (12.5)	3 (10.7)	0.608
Pre-dilation size (mm)	19.0 ± 1.7	19.3 ± 1.8	18.8 ± 1.6	0.431
PVL at discharge				0.237
Mild, (*n*, %)	24 (54.5)	7 (41.2)	17 (63.0)	
Mild-moderate, (*n*, %)	12 (27.3)	5 (29.4)	7 (25.9)	
Moderate, (*n*, %)	8 (18.2)	5 (29.4)	3 (11.1)	
Severe, (*n*, %)	0 (0.0)	0 (0.0)	0 (0.0)	
Medications at discharge				
Beta-blocker (*n*, %)	6 (13.6)	1 (5.9)	5 (18.5)	0.154
Diuretics (*n*, %)	14 (31.8)	4 (23.5)	10 (37.0)	0.509
ACEi/ARB (*n*, %)	16 (36.4)	6 (35.3)	10 (37.0)	0.907

BMI, body mass index; CKD, chronic kidney disease; LEAD, lower extremity artery disease; eGFR, estimated glomerular filtration rate; BNP, B-type natriuretic peptide; LVEF, left ventricular ejection fraction; LVOT, left ventricular outflow tract; PVL, paravalvular leakage; Ca-Vol, calcium volume; ACEi, angiotensin-converting enzyme inhibitor; ARB, angiotensin II receptor blocker

^a^Median (interquartile range)

## Discussion

In the present study, we evaluated the relationship between SEVs expansion and PVL in the chronic phase. We found that Evolut PRO/PRO+ and FX valves expanded in the chronic phase in all patients, and PVL decreased to one-third of the total. To the best of our knowledge, the present study is the first to show that SEVs expand in the chronic phase by CT scan.

SEVs are used in cases of severe aortic valve calcification due to the high risk of aortic annulus rupture [5]. On the other hand, PVL remains a problem that affects long-term mortality after TAVI [4]. As TAVI has expanded to younger patients and those at low surgical risk [1, 2], PVL is a more important issue in TAVI patients.

In addition, there is a study comparing transcatheter heart valve (THV) diameters immediately after THV deployment and at the end of the procedure, showing larger THV diameters at the end of the procedure [8]. However, the expansion of SEVs over time and the contribution of valve expansion to the PVL improvement in the chronic phase were not assessed. Moreover, a previous study has reported that PVL improves within 1 year after TAVI using SEVs [9], but the mechanism of reduction of PVL has not been investigated. We have illustrated that in cases of severe aortic valve calcification, even if PVL remains immediately after TAVI using SEVs, it can improve with valve expansion during the follow-up period. Our study showed that the cases without PVL improvement had minimal PVL immediately after TAVI. On the other hand, the PVL-improved group had more PVL immediately after TAVI. Considering the potential reduction of PVL in the chronic phase after SEV implantation, a treatment strategy of not performing post-dilation for PVL may also be acceptable given the risk of annular rupture associated with balloon dilation.

In summary, the present study demonstrated valve expansion in all cases, but PVL decreased to one-third of the total in the remote period after TAVI using SEVs. However, there is two-thirds of all cases in which PVL did not decrease despite valve enlargement, making it necessary to consider other mechanisms for the decrease of PVL.

In this study, the expansion of the valve was evaluated at the basal area, and it is considered that the expansion of the basal area reflects the overall expansion of the valve. The expansion of the entire valve increased the vertical axis contact area, which may lead to the suppression of PVL. In addition, possible explanations for this regression of PVL are that the continuous outward expansion of nitinol frames may reduce paravalvular gap to allow more tissue growth in chronic phase.

This study demonstrated the association between valve expansion of SEVs and PVL decrease during the follow-up period. This finding emphasizes the importance of considering SEVs in patients with severe aortic valve calcification to reduce the risk of aortic annular rupture. PVL remains a serious problem affecting the clinical outcomes of TAVI patients. In patients with severe aortic valve calcification, SEVs are chosen to avoid aortic annulus rupture. Even if there is significant PVL immediately after TAVI, valve expansion, and PVL improvement could be observed in the chronic phase, potentially improving patient prognosis in the long-term. Valve expansion and PVL improvement overtime may contribute to better patient outcomes. However, some cases in this study did not reduce PVL despite valve expansion, emphasizing the need for further research into other mechanisms of PVL reduction and continued improvements in valve design. The treatment options for AS are varied and the present study may assist in the choice between TAVI and surgical aortic valve replacement, as well as the selection of SEVs or BEVs in TAVI.

### Study limitations

The present study has several limitations. First, this was a single-site study with a small sample size. Second, patients with bicuspid valves and TAVI in surgical aortic valve replacement cases were excluded from this study due to the differences in the valve structure. Third, our study population was skewed toward patients with severe calcification and a high risk of aortic root rupture. Nevertheless, this reflects the actual clinical practice of opting for SEVs in cases where there is a significant risk of rupture. To confirm our findings, further investigations of TAVI using SEVs with a larger sample size and a longer follow-up period are needed.

## Conclusions

Our study demonstrated that SEVs expand in the chronic phase after TAVI and could contribute to PVL reduction.

## Data Availability

Data is available.
